# Social Contexts of Subjective Aging Perceptions

**DOI:** 10.1093/geroni/igaa057.2108

**Published:** 2020-12-16

**Authors:** Ella Cohn-Schwartz, Liat Ayalon

## Abstract

The way adults perceive their aging process is an important predictor of later life outcomes, including mental and physical health. Despite the importance of living a socially active life in old age, the inter-connections of individuals’ perceptions of aging with their social lives and behaviors are not well-understood. This symposium addresses questions of how the social environment and social behaviors are related to subjective aging perceptions, including subjective age and self-perceptions of aging. Two papers examine self-perceptions of aging in the context of couple relations. Mejía and colleagues focus on married older adults’ shared beliefs about aging, showing that within older couples, beliefs about aging are shaped in part through partners’ co-experience of each other’s biological aging. Kim and colleagues also examine couples, finding evidence that changes across time, as well as average differences in individual characteristics, may affect self-perceptions of married/partnered men and women differently. The final two papers examine the interplay between chronological age and perceptions of aging. Weiss and Weiss examine the social conditions and consequences of subjective age across the life span in the work domain, demonstrating that feeling relatively older among young adults and younger among older adults predicts proactive behaviors such as speaking up. Cohn-Schwartz and colleagues investigate the bi-directional temporal associations of adults’ self-perceptions of aging and the age composition of their social networks. The symposium concludes with summarizing remarks from the discussant who will suggest possible directions for future research on the social contexts of the perceived experience of aging.

